# Supplementary material for: Are carbon emissions trading and green financial instruments synergistic? -Comprehensive quantitative research based on content analysis
Source: PLoS One. 2024 Mar 7;19(3):e0298601. doi: 10.1371/journal.pone.0298601 (PMC10919601; doi:10.1371/journal.pone.0298601)
Supplement: S1 Data — (ZIP) [file pone.0298601.s001.zip › Data/地级市Z_F工作报告.zip!/╣≤╓▌/╣≤╤⌠╩╨/└┤╫╘╣1⁄2╓┌║┼í╛┬φ┐╦╩2╛▌═°í┐_2011.docx]

各位代表：

现在，我代表市人民政府向大会作政府工作报告，请连同《贵阳市国民经济和社会发展第十二个五年规划纲要（草案）》一并审议，并请市政协各位委员和列席人员提出意见。

一、“十一五”时期国民经济和社会发展的回顾

过去五年，是我市发展进程中极不平凡的五年。全市人民在省委、省政府和市委的坚强领导下，在市人大、市政协的监督、支持和帮助下，坚持以邓小平理论和“三个代表”重要思想为指导，深入贯彻落实科学发展观，锐意进取、艰苦奋斗，积极应对国际金融危机的严重冲击，克服百年不遇的特大雪凝灾害和特大旱灾造成的不利影响，全面完成“十一五”规划的各项目标任务，经济又好又快发展，社会和谐稳定，人民生活持续改善，生态文明城市建设向纵深推进，为“十二五”时期加速发展、加快转型、推动跨越打下了坚实的基础。

（一）这是经济发展最快、质量效益最好、综合竞争力极大提升的五年

经济实力明显增强。生产总值从2005年的525.62亿元增加到2010年的1121.82亿元，增长了1.1倍，年均增长14.2%，比“十五”快1个百分点，高于全国、全省平均增速，创建国以来用5年时间翻番的最快增速。其中三次产业年均分别增长7.9%、12.2%和16.9%。人均生产总值突破4000美元。财政总收入从133.15亿元增加到304.64亿元，年均增长18%；地方财政收入从49.8亿元增加到136.3亿元，年均增长22.3%，分别比“十五”快1.3、3.8个百分点。固定资产投资累计完成3317.51亿元，年均增长24.3%。城市居民人均可支配收入、农民人均纯收入年均实际分别增长7.9%、9.7%，农民人均纯收入2008年首次超过全国平均水平。进出口总额从11.36亿美元增加到22.75亿美元，增长1倍。县域经济发展壮大，建制区（市、县）全部进入经济强县行列。2010年末金融机构存、贷款余额分别达3054.07亿元和2588.73亿元，是2005年末的2.4倍和2.5倍。经过5年的艰苦奋斗，我市生产总值、固定资产投资、工业总产值均突破千亿元，主要经济指标超过兰州，在西部省会城市“进位”，部分重要监测指标达到全面小康社会标准，站在科学发展新的战略起点上。中国社科院发布的《全球城市竞争力报告（2009—2010）》显示，在全球500个城市中，贵阳名列综合竞争力上升最快城市的第4位。

城市核心竞争力进一步增强。市级应用技术研究与开发资金投入占财政经常性支出的比重由2.59%增加到2.9%，实施科技计划项目1557个；建成2个国家级工程技术研究中心、2个国家级重点实验室，以及一批省级工程技术中心、重点实验室，形成产学研结合紧密、成果转化加快的城市创新体系。新增中国驰名商标12件，专利申请量、授权量分别达10666件、6431件。连续五届获得全国科技进步考核先进城市称号，被确定为国家级创新型试点城市、国家知识产权工作示范城市。

城市知名度、美誉度显著提高。先后获得中国优秀旅游城市、国家森林城市、全国绿化模范城市、中国人居环境范例城市、国家园林城市、中国避暑之都、全国双拥模范城等称号；连续三次被评为全国创建文明城市工作先进城市。被国家统计局评为建国60年最具代表性的60个城市之一，排在第36位。

（二）这是工业做大做强、服务业增速增比、农业做特做优的五年

工业经济持续快速增长。规模以上工业总产值从574.06亿元增加到1058.15亿元，年均增长12.1%。结构进一步优化。装备制造、现代制药、磷煤化工、铝及铝加工、卷烟与特色食品等五大产业总产值达752.9亿元，占规模以上工业总产值比重达71.1%；装备制造业、现代制药、磷煤化工、卷烟与特色食品业总产值均突破100亿元。高新技术产业总产值达233.3亿元，占规模以上工业比重22%。规模以上工业企业达603户，产值超亿元企业从88户增加到157户，超10亿元企业从8户增加到12户。效益大幅提升。规模以上工业企业利润总额从16.39亿元增加到56.56亿元，年均增长28.1%。聚集发展格局初显。成立10个副县级园区办公室（管委会），强力推进工业园区建设，麦架——沙文生态工业园、小河——孟关装备制造业生态工业园“八通一平”建设进展顺利，龙洞堡食品轻工业园和贵州百灵苗药工业园等园区加快推进，“十大”园区相继开工50余个项目，入驻企业83户。发展后劲极大增强。工业投资累计达930.89亿元，年均增长20.8%；开磷集团120万吨磷铵、贵阳卷烟厂异地搬迁等一批重点项目建成投产，中航飞机发动机生产线、首钢贵阳特殊钢生产基地等投资30亿元以上的重点项目建设进度加快，息烽循环经济精细磷煤化工工业园年产30万吨硝基复合肥、江苏雨润冷鲜食品加工等一批大项目、好项目开工建设。

服务业长足发展。被确定为首批国家服务业综合改革试点城市。第三产业增加值达607.76亿元，占全市生产总值的比重从45.9%提高到54.2%。旅游业给力领跑。召开四次旅游发展大会，青岩古镇、南江峡谷、阳明文化园等景区知名度大幅提升，成功推出修文野生动物园、开阳十里画廊、泉城五韵等新景点，“中国避暑之都”、“爽爽的贵阳”城市旅游品牌效应凸显。新增国家4A级景区6个，星级以上酒店达到55家，建成和在建五星级酒店达15家，酒店年接待人数超过600万人次，实现由全省旅游集散中心向全国重要旅游目的地、西南旅游服务中心的重大转变，旅游总收入从60.38亿元增加到425.96亿元。“引金入筑”工程取得实效。开工建设贵阳国际金融中心，成功引进花旗银行、中信银行、浦发银行、招商银行等国内外知名银行在我市开设分支机构；银河证券、华泰联合证券以及人寿保险公司等保险、证券公司获批筹建分支机构。现代物流业加快发展。清镇物流园区、西南物流中心、扎佐物流园、二戈寨物流园等项目加快建设，启动贵阳（中国）西南国际商贸物流城建设和五里冲农产品批发市场、花溪大道汽车市场带搬迁，荣获“物流中心城市最佳投资环境奖”，被列为“全国流通领域现代物流示范城市”。商贸流通业持续繁荣，社会消费品零售总额年均增长18.9%。会展业取得突破。建成西部规模最大、功能最全、设施最先进的国际会展中心，生态文明贵阳会议、亚洲青年动漫大赛、医药博览会等成为全国知名会展活动，荣获“中国绿色会议城市”称号，为打造“中国夏季会展名城”夯实基础。软件和动漫产业初具影响。建立数字内容产业园，朗玛科技、青年影视等动漫企业崭露头角，产值年均增长50%以上。

农业产业化水平大幅提升。畜、禽、蛋、奶、果、蔬、花、药、茶等特色优势产业加快发展，温氏、南江、好一多、三联、台农等龙头企业发展壮大，产值上亿元龙头企业达15家；改变鸡蛋、蔬菜内供不足靠外运的状况，市场供应能力能满足每个市民每天一只鸡蛋、一杯牛奶，贵阳花卉、“黔山牌”蔬菜等远销国内外市场，市民“米袋子”、“菜篮子”、“油罐子”、“奶瓶子”极大充实。切实推进城乡一体化，加快新农村建设，持之以恒抓县乡公路改造、解决农村饮水安全、病险水库治理以及通村公路、串户路、农田基本建设，极大地改善了农村生产生活条件。

（三）这是城市拓展最快、市容市貌明显改善、宜居宜业宜游城市建设取得突出成效的五年

大力实施城市拓展计划，城市进入大扩容、大聚集、大跨越的新时期。城建投资累计完成1366.5亿元，占全部投资的41.2%，是“十五”时期的2.64倍。坚持规划先行。高质量完成《贵阳市城市总体规划（2010—2020年）》、《贵阳市土地利用总体规划（2006—2020年）》修编工作，中心城区规划面积扩展到1230平方公里，城市空间布局形态由“双中心、多组团”向“一城三带多组团、山水林城相融合”转换。大手笔推进路网建设支撑城市大扩容。实施“畅通工程”，建成环城高速公路、黔灵山路、甲秀南路、北京西路、机场路、水东路、朝阳洞路、市北路、宅吉路、贵惠路、开发大道、黔江路、珠江路、金朱路、长岭路、观山西路二期等主干道和一批支路，建成西南地区最大铁路编组站——贵阳铁路枢纽贵阳南编组站。配合抓好贵阳至广州、成都、昆明、长沙、重庆等高速铁路、客运专线等建设，加快建设市域快速铁路、城市轨道交通和“两路二环”，城市发展框架进一步拉开。以城市综合体为细胞加快城市聚集。建设了世纪金源、香港新世界、保利温泉新城和山水黔城等大型城市综合体。加快麦架——沙文——扎佐片区基础设施建设和产业聚集，推动高新技术产业“北拓”；推进高等教育聚集区、花溪湿地公园、小河——孟关装备制造业工业园建设，通过“南延”加快花溪、小河等区域城市化进程和产业聚集；加快三马片区物流商贸带、金阳客车站、火车北站等建设，配套观山湖公园、奥体中心、贵阳大剧院、市美术馆、市民健身中心、金阳医院、青少年和妇女儿童活动中心等公建设施，推动盘江煤电、开磷集团等总部大楼的建设，通过“西连”三马片区、清镇市促进老城区与金阳新区融合发展，金阳新区人气、商气大幅聚集，初具现代化城区格局。整体启动龙洞堡——小碧——永乐片区建设，抓好龙洞堡食品轻工业园、渔安安井十里花滩建设，推进“东扩”建设东部新城和新天组团。

大力加强城市精细化管理，城市品质大幅提升。相继开展“为民办实事、满意到万家”、“整脏治乱”、“四城同创”、“三创一办”等活动。大力推进“数字贵阳”建设，数字化城市管理系统投入使用，城市社会治安、交通管理事件等即时处置率从50%提高到90%以上。实施主城区主干道白改黑、环岛改造等工程，建成中华路等13个地下人行通道及一批人行天桥，完成40余公里综合管网入地、人行道整治、房屋立面整治工程。切实抓好城市厕所文明、菜场文明、垃圾文明、无障碍文明，取缔延安路、青云路等夜市，规范占道经营，完成新增出租车及降低出租车起步价、搬迁中心城区客车站等工作，政府投资建设的523个公厕全部免费开放，建成了以25万吨污水处理为标志的新庄污水处理厂一期和小河污水处理厂二期等城市污水处理设施，逐步推开城市主干道机械清扫和生活垃圾分类试点工作，完成177个农贸市场升级改造，成功创建“全国无障碍城市”和“节水型城市”。城市面貌发生翻天覆地变化，整洁、靓丽、有序，品位实现大提升。

大力开展生态建设，城市环境极大改善。累计安排逾4000万元市级专项资金引导发展，实施95个循环经济项目，新上项目中磷矿资源就地转化率达到100%，现有企业磷、铝资源转化率分别达60%、78.5%，循环经济工作走向全国前列。城区清洁能源普及率达98.5%，建成22万口沼气池，惠及农村百万人口。关停、搬迁贵州水泥厂等一批重点污染源。单位GDP能耗和单位工业增加值能耗均控制在省下达指标内；2009年提前完成“十一五”二氧化硫排放量减排任务。建立生态补偿机制；实施天然林保护、石漠化综合治理、退耕还林成果巩固等国家重点工程，打好“森林保卫战”、“治理两湖一库”等硬仗。实现“两湖一库”水质明显好转的阶段性目标，森林覆盖率年均提高1个百分点，人均公共绿地面积从8.31平方米提高到9.86平方米，城区空气质量优良率稳定在95%左右。被确定为全国生态文明建设试点城市、国家首批低碳城市试点。

（四）这是民生投入最大、惠及民生最广、人民群众生活水平极大提高的五年

扎实推进“六有”民生行动计划，让发展成果更多地惠及全市人民。财政用于民生的投入累计达379.29亿元，占财政支出的52.1%。每年坚持为民办好“十件实事”，累计投入资金达222.66亿元。学有所教方面：义务教育“两免一补”、进城务工人员子女接受义务教育、对市属中等职业学校本市户籍学生免除学费、高中阶段免费教育试点等方面均走在了全国前列，“两基”工作顺利通过“国检”，建成贵阳一中、实验三中和贵阳学院、护理职业学院、职业技术学院，启动高等教育聚集区、职业教育聚集区建设。获得全国首届地方教育制度创新贡献奖。劳有所得方面：以创业带动就业，突出抓好高校毕业生、农民工、就业困难群体“三大重点人群”就业工作，52%的社区成为充分就业社区，率先实现“零就业家庭”动态为零，累计新增城镇就业26.02万人，农村劳动力转移就业13.42万人，城镇登记失业率控制在4%以内。被列为国家创业型试点城市。病有所医方面：新型农村合作医疗参合率达97%以上；城镇职工基本医疗保险参保率达90%以上，参保人员个人负担比例比2005年降低10个百分点，低于全国平均水平13个百分点；惠民医疗政策、医疗救助制度100%覆盖城乡困难群众。老有所养方面：率先建立新型农村社会养老保险和被征地农民必须参加社会养老保险等保障制度和政策，城镇职工基本养老保险、新型农村社会养老保险、工伤保险、生育保险参保人数分别达72.75万、64.8万、55.37万、80.65万人。住有所居方面：建立健全以廉租住房为重点、多渠道解决城乡低收入群体住房困难的政策体系，建成廉租住房40万平方米，将22630户人均收入在975元以下、居住面积在15平方米以下的城市低收入家庭全部纳入保障范围，实现应保尽保；在全省率先完成农村茅草房和危房改造任务，104728户农村家庭圆了安居梦。居有所安方面：持续深入开展严打两抢一盗、打黑除恶、禁毒等专项行动，群众社会治安满意度和安全感增强，获得“全国社会治安综合治理优秀城市”和“长安杯”，息烽县成为“无毒县”。强化矛盾纠纷排查调处，“清积案、促和谐”工作成效显著。人口计生工作连续16年保持全省目标考核一等奖第一名，被确定为全国人口计生综合改革示范市、全国流动人口计划生育基本公共服务均等化试点市。连续多年保持全省安全生产工作一等奖的好成绩。文化体制改革迈出实质性步伐，组建贵阳日报传媒集团、贵阳演艺集团，贵阳交响乐团是全国第一家民营资本投入的城市职业交响乐团。积极调动社会力量参与民生事业，大力传播慈善文化，组建“绿丝带”志愿者队伍，志愿者达到36万人，形成互帮互助、服务社会的文明风尚。应急、审计、监察、仲裁、气象、物价、人事、民族、宗教、人防、移民、外事侨务、对台、市志、档案、妇女儿童、老龄等其他各项工作取得新成绩。

2010年，全市经济实现又好又快发展。生产总值增长143%。财政总收入增长20.9%，地方财政收入增长29.4%。全社会固定资产投资完成1019.31亿元，增长30.2%。社会消费品零售总额完成484.78亿元，增长19.6%。城市居民人均可支配收入达16597元，增长10.3%；农民人均纯收入达5976元，增长12.4%。引进内资实际到位资金453.76亿元，增长23.1%；实际利用外资1.35亿美元，增长20.1%。

各位代表，回顾五年来取得的巨大成就，我们有多方面深刻体会，主要是：一是必须坚持加快发展、科学发展、率先发展。我们始终坚持将发展作为解决贵阳所有问题的关键，努力解决“慢”这个主要矛盾，将中央和省委、省政府“作表率、走前列、做贡献”的要求与贵阳实际结合起来，只要符合科学发展观的要求，有条件、有效益，就努力加快发展。二是更加注重统筹人与自然和谐发展、统筹城乡发展、统筹经济社会发展。以生态文明理念引领工业发展，力求以更小的投入、更低的消耗，创造更大的效益，走出新型工业化道路。坚持城乡一体化发展，扎实推进人口向城镇集中、产业向园区集中、土地向集约经营集中。在推动经济更好更快发展的同时，更加注重社会建设，促进经济社会全面、协调、可持续发展。三是加快推进创新驱动、开放带动、改革促动。以创新为城市发展之魂，实施科教兴市和人才强市战略，切实把经济发展转变到依靠科技进步和提高劳动者素质上来。树立开放理念，加快形成全方位、宽领域、多层次的开放格局。把改革的力度、发展的速度和社会可承受的程度更好地统一起来，为加快发展提供体制机制保障。四是尽心竭力为民、利民、惠民。坚持“人民群众生活中的小事、就是政府工作中的大事；人民群众生活中的难点，就是政府工作的重点”，切实做到“民有所呼、我有所应”，让更多人共享改革发展成果，让全体市民过上更加幸福安康的生活。五是抓好法治政府、服务型政府、廉洁政府建设。坚持依法行政，自觉接受市人大及其常委会的法律监督、工作监督和市政协的民主监督，听取各民主党派、工商联和无党派人士的建议，法治政府建设上台阶。推进行政审批制度改革，成为省会城市中行政审批事项最少、效率最高的城市之一；强化财政预算管理，连续四年实现公用经费支出“零增长”；推行政务公开，政府透明度在43个省会城市及较大城市中位列第7位，政务服务效能和水平上台阶。加强行政监察和审计监督，坚持不懈地开展反腐败斗争和政风建设，廉洁政府建设取得新成绩。

各位代表，“十一五”时期我市经济社会发展取得辉煌成就，成绩来之不易。这是省委、省政府和市委正确领导的结果，是在历届市委、市政府坚持“作表率、走前列、做贡献”，不懈努力奋斗，为我们打下的坚实基础上取得的结果，是市人大、市政协认真监督、大力支持的结果，是全市干部群众努力奋斗、辛勤劳动的结果。在此，我代表市人民政府，向全市各族人民，向各位人大代表和政协委员，向各民主党派、工商联、人民团体和各界人士，向中央及省在筑单位，向驻筑人民解放军、武警官兵和公安政法干警，向所有关心和支持贵阳建设与发展的各界朋友，表示衷心的感谢，并致以崇高的敬意！

在充分肯定成就的同时，我们也清醒地看到，我市经济社会发展中还存在许多困难和问题，主要表现在：发展速度不够快，综合实力不强；产业结构不尽合理，资源依赖程度高，科技支撑能力不强；城乡居民收入增长与经济增长不同步，城乡发展不平衡依然比较突出，老百姓反映强烈的交通拥堵、就学就医等问题仍未从根本上得到解决，处于社会矛盾多发期，而且财政处于偿债高峰期，制约科学发展的体制机制性障碍依然较多。对这些问题，我们将采取更为有效的措施认真加以解决。

二、“十二五”时期国民经济和社会发展的展望

“十二五”规划编制工作是在市委、市政府领导下进行的。2009年初，市委、市政府组织有关方面，对全市经济社会发展面临的重大问题进行专题研究，市人大、市政协也就“十二五”发展的一系列问题组织开展调研，为编制《纲要（草案）》提供了重要基础。市委八届十次全体（扩大）会议后，市政府根据《中共贵阳市委关于制定国民经济和社会发展第十二个五年规划的建议》精神，深入研究和编制“十二五”规划纲要，组织各方面专家进行咨询论证，在全市范围内广泛征求意见。《纲要（草案）》的编制过程，是深化市情认识、总结发展经验、把握未来趋势的过程，是发扬民主、集思广益、科学决策的过程，是增强信心、凝心聚力、共谋发展的过程。

《纲要（草案）》根据市委《建议》，提出“十二五”时期贵阳经济社会发展的总体思路：高举中国特色社会主义伟大旗帜，以邓小平理论和“三个代表”重要思想为指导，深入贯彻落实科学发展观，顺应人民群众过上更好生活的新期待，始终秉持为人民谋幸福的理念，以加快转变经济发展方式为主线，按照“作表率、走前列、做贡献”的要求，牢牢把握加速发展、加快转型、推动跨越的主基调，遵循“走科学发展路，建生态文明市”的基本路径，突出实施工业强市战略和城镇化带动战略，以扩大投资为重要举措，以三次产业提速增效为主攻方向，以科技和人才为核心支撑，以生态建设和环境保护为基本前提，以改革开放为强劲动力，以保障和改善民生为根本目的，当好全省经济社会发展的“火车头”、黔中经济区崛起的“发动机”，在全省率先全面建设小康社会，提升全体市民的幸福指数，实现经济社会发展的历史性跨越。

我们将按照《纲要（草案）》，强化三方面工作：

(一)率先全面建设小康社会，当好全省发展的“火车头”

一是经济实力更强。工业化、城镇化水平明显提升，现代服务业加速发展，六大支柱产业更加壮大，生态农业长足发展，自主创新能力明显增强，初步形成城乡协调发展新格局。地区生产总值年均增长15%，一般预算收入年均增长17%。力争四年、确保五年实现地区生产总值和财政总收入翻番，分别超过2000亿元、600亿元，人均生产总值超过6000美元。全社会R&D占GDP比重和科技进步贡献率逐年提高。社会消费品零售总额年均增长18%。

二是幸福指数更高。“十大民生工程”扎实推进，科技、教育、文化、卫生、体育等各项社会事业快速发展，覆盖城乡居民的基本公共服务体系更加完善，城乡文明程度明显提升，社会更加和谐稳定，市民满意度和幸福感切实增强。城乡居民收入与地区生产总值同步增长，力争达到西部省会城市平均水平，城市居民人均可支配收入达3.2万元，农民人均纯收入达1.2万元。物价总水平保持基本稳定。城镇新增就业35万人，城镇登记失业率控制在4.5%以内，保持“零就业”家庭动态为零；人均受教育年限达11年；人均住房面积达30平方米；人均期望寿命达74岁；各项卫生指标达到西部省会城市的平均水平，千人拥有卫生技术人员6人；人口自然增长率控制在56‰以内。

三是生态环境更好。有效降低资源消耗，有效治理环境污染，生态环境质量明显改善，生态文明建设试点城市和低碳城市试点工作取得重大进展。清洁能源使用率提高到60%；主要污染物排放总量控制在国家和省的要求以内，空气质量优良率达95%；城市生活污水集中处理率达95%以上；森林覆盖率达45%。

（二）加快新型工业化、城镇化和农业产业化步伐，做黔中经济区崛起的“发动机”

一是强力推进新型工业化，加快建设现代产业体系。实施“千亿元产业”行动计划，力争把装备制造业、磷煤化工业、医药保健业培育成千亿元产业，将铝及铝加工、特色食品等产业培育成500亿元产业，明显提高新能源、新材料、航天航空、节能环保、新一代信息技术等战略性新兴产业比重，以支柱产业、新兴产业带动、支撑大发展。实施“百亿元企业”行动计划，力争培育4个年销售收入超百亿元企业，培育8个年销售收入超50亿元企业和一大批年销售收入超10亿元以上企业。加快工业园区建设，逐步形成产业分布合理、特色突出、功能完善、有较强配套能力的产业园区。力争工业总产值突破2500亿元，实现从工业化中期向后期的跨越。抓住我市列为国家服务业综合改革试点城市的机遇，实施“服务业跨越千亿元”计划，将我市建成具有国际影响力的旅游避暑城市和夏季会展名城，国内重要的区域性物流中心、商贸中心、金融中心。

二是加强城镇规划建设，加快建设新型城镇体系。积极参与编制《黔中城镇群规划》、《黔中经济区发展规划》，重点编制好综合交通体系、重点功能区、产业布局等专项规划，在更大空间、更高起点上谋划贵阳新发展。以城镇规划为龙头，推进城乡规划全覆盖，重点规划、建成一批10万人聚集的功能小区。继续实施“北拓、南延、西连、东扩”城市拓展计划，以老城区、金阳新区为核心，连片发展小河、二戈寨、三桥马王庙、甘荫塘、花溪、白云、清镇、新天等区域，优化城市空间布局。按照“中心放射型”的城镇体系空间布局，高标准建设沿贵黄、贵遵、贵毕、贵开、贵惠、厦蓉、市域快速铁路、环城高速公路等重要交通干线的城镇发展走廊，推进龙洞堡与龙里联动发展以及一批重点城镇和特色城镇建设。城镇化率达75%以上。

三是推进农业大发展、大调整，构建现代生态农业产业体系。围绕“畜、禽、蛋、奶、蔬、果、花、茶、药、烟”等特色优势产业，大规模、高标准调整农业产业结构，发展农业产业化，培育龙头企业，促进农业增效、农民增收。加大公共财政向三农倾斜、基础设施向三农延伸、公共服务向三农覆盖力度，尽快形成安全、完善的城乡生产、生活用水体系，加快供电、公交、通信、电视、污水和垃圾处理网络化建设，促进中小城镇与城市顺畅连通。继续深化户籍制度改革，推进农村土地承包经营权流转，形成有利于农民下山、进城进镇的体制机制，促进“大城市、大农村”向城乡一体化迈进。

（三）加强项目建设、环境建设，全力加速发展、加快转型、推动跨越

一是加强项目建设，以大投资促大发展、快发展。狠抓项目储备，高效率促进项目建设。确保固定资产投资累计超过10000亿元，年均增长30%。把产业投资作为重中之重，以优化投资结构促进产业结构、经济结构优化升级。确保工业投资累计达到3000亿元以上，力争达到5000亿元。

二是加强环境建设，以大开放促大发展、快发展。加强以交通为重点的基础设施建设，配合抓好贵阳通往全国快速铁路网和龙洞堡机场改扩建等工程，建成市域快速铁路、城市轨道交通1、2号线和“三条环线十六条射线”骨干路网，形成完善、快捷的现代城市综合交通体系。实现铁路交通迈进“高铁”时代，航空与全国省会城市直航，推动贵阳由节点型城市向枢纽型城市跨越。坚持以招商引资和项目建设为生命线、服务企业为本职，着力营造项目引进建设环境、企业生产经营环境、政府管理服务环境。

三、2011年经济社会发展的主要目标和任务

今年将迎来建党90周年，是“十二五”规划的起步之年，做好全年各项工作，具有十分重要的意义。我们既要承载全国各族人民的重托，全力以赴协办好全国少数民族传统体育运动会，展示各民族和谐共处、团结奋进的形象，提升贵州、贵阳的知名度、影响力；又要按照又好又快、更好更快发展的要求，确保“十二五”开好局、起好步。今年经济社会发展主要预期目标：生产总值增长17%，其中第一产业增长13%，第二产业增长20%（其中规模以上工业增加值增长20%），第三产业增长15%。财政总收入增长17%；地方财政收入增长17%。全社会固定资产投资增长40%。社会消费品零售总额增长19%。实际直接利用外资增长30%。外贸进出口总额增长17%。城市居民人均可支配收入增长15%；农民人均纯收入增长15%。城镇登记失业率控制在4.5%以内。人口自然增长率控制在6‰以内。单位生产总值综合能耗、主要污染物排放总量控制在省下达指标内。

为民办的十件实事：

新建和改扩建公办幼儿园40所和小学、初中各11所，建成金阳高中，启动高等教育聚集区、职业教育聚集区基础设施建设，完成贵阳幼儿师范学校升格为幼儿师范高等专科学校的筹建。

实现城镇新增就业6万人，保持“零就业”家庭动态为零，确保城镇基本养老保险扩面5万人、城镇基本医疗保险扩面4万人。

完成农村村寨串户路建设1000公里、村寨清洁工程20个、大中型沼气工程70个，新建500亩高标准连片蔬菜种植基地12个（三县一市两郊区），确保“菜篮子”供应。

新开工建设保障性住房70万平方米，竣工保障性住房20万平方米，将人均住房建筑面积不足15平方米的城市低收入家庭纳入住房保障范围。

开工建设贵阳客车站、盐沙路、开息路、桐荫路、东站路、富源南路；建成贵阳客运东站、金清线、宾阳大道北段，力争建成白修线；新增一批公交大巴车、1000辆出租车，一批迷你巴士，开辟公交线路到住宅小区，解决1公里断头路出行难问题。

改造城区背街小巷破损道路30条，新建或改造公厕20座、垃圾中转站20座，更换社区密闭式垃圾收运车200辆，新建金阳新区人行地下通道4座，改扩建老城区人行过街系统4处。

免费开放观山湖公园，建成花溪湿地公园，开工建设中央生态公园（原贵阳一中旧址），新建山体公园健身步道50公里，开放奥体中心。

完成41个乡镇、47个街道文化信息资源共享工程基层服务点建设，修缮维护贵阳以珍珠泉、圣泉为代表的21口古井及贵阳古八景。

开工建设市未成年人保护中心、市社会福利院“老人康复楼”、市精神病医院和门诊大楼，建成3个县级医院、5个中心乡镇卫生院、18个社区卫生服务中心，开展免费提供基本殡葬服务试点工作。

完善治安卡点300个，进一步推进小区技防工程建设，增加交警协勤人员500名，购置警用直升机一架、超高层云梯消防车一台。

围绕以上目标，我们将认真做好以下六个方面工作：

（一）奋力实施“千亿元产业、百亿元企业”行动计划，在三次产业提速增效、协调发展、增强竞争力上求突破、见实效

坚持“工业强市”战略，推动工业经济持续快速增长。实施工业投资倍增计划。以开展“项目大推进年”为契机，切实落实全省工业发展大会、全市工业振兴大会的精神，发挥重大项目服务工作队的协调服务作用，集中力量解决工业投资长期不足的问题。除现有工业发展资金外，新安排装备制造业、磷煤化工业、医药产业发展专项资金，争取上级配套资金支持设立高新技术与战略性新兴产业投资基金。以高新技术开发区、经济技术开发区及各区（市、县）为责任主体，以各工业园区、产业投融资平台为主要载体，加大企业投资。实现工业投资增速高于基础设施投资，新增建设用地重点向工业项目倾斜，使工业投资增长100%以上（含园区基础设施），占全社会固定资产投资总额的30%左右。启动千亿元产业行动计划。加快首钢贵钢异地搬迁、中航发动机项目、贵州轮胎1280万条子午线胎异地技改、詹阳重工异地搬迁、普天异地搬迁、险峰异地搬迁等项目建设，开工建设中国普天新能源及现代物流装备产业基地、南方汇通轨道车辆制造等项目，提高装备制造业产能和集聚发展水平，装备制造业投资超过100亿元。建成开阳化工50万吨合成氨、贵州广铝年产80万吨氧化铝一期，加快贵州中铝公司年产15万吨铝板带、开磷800万吨绿色矿山、安达30万吨有机膦及10万吨新能源材料、剑化异地搬迁、水晶公司改扩建、中铝贵州分公司煤电铝联营等项目建设，磷煤化工、铝加工产业投资分别达20亿元、30亿元。开工建设益佰工业园、百灵苗药工业园、百强药品生产工业园、黔峰生物科技园、圣济堂GMP改造等项目，加大贵酒、雨润、康师傅、统一食品等项目推进力度，医药及保健品、食品产业投资力争达到30亿元。启动百亿元企业行动计划。按照“大企业、大产品、大品牌”思路，支持开磷集团、贵航集团、061基地、贵州轮胎公司、中铝贵州分公司、中烟贵州公司、中电振华、益佰集团、神奇制药、圣济堂公司、老干妈公司、贵阳海信、华能焦化、险峰机床等骨干企业壮大规模，提升实力。对重点培育企业在争取国家重大专项、技改资金、项目贴息、人才引进、贸易方面制定优惠政策，培育2家产值超100亿元的龙头企业。加快工业园区建设和集聚发展。实施产业园区控规及产业规划，完成“十大”园区骨干路网建设，水、电、通信等基础设施基本满足企业入驻条件。以投融资平台公司为载体，以市级配套及贴息资金引导、带动各级各类投资，使园区基础设施投资达200亿元，建成标准厂房10万平方米以上，园区共新引进项目120个。鼓励“园中园”、特色产业基地率先发展，高新区新天园区、金阳园区、中电振华新材料新能源产业基地和白云铝及铝加工基地、小河军民结合装备制造业基地、老干妈云关园区、开阳和息烽循环经济磷煤化工园区等配套基本成熟的园区工业增加值增长25%以上，全部工业园区产值有大的提高。

加快发展现代服务业，扎实推进区域性服务中心建设。壮大提升旅游业。以在花溪召开第五届旅游产业发展大会为契机，继续举办避暑季、温泉季系列活动，加快花溪湿地公园、乐湾国际温泉、乌当生态体育公园、白云长坡岭森林公园体育休闲等项目建设和天河潭、南江大峡谷等5A级景区创建进程；继续举办森林音乐会，重点打造1—2个具有较大影响力的文化旅游产品；建设1—2个4A级旅游景区游客服务中心体系，加快香格里拉、万豪、洲际、温德姆等品牌酒店建设，力争2—4家五星级酒店开业。旅游人数增长20%，旅游收入增长30%。加快发展现代物流业。重点抓好贵阳（中国）西南国际商贸物流城和扎佐、清镇等物流园区、改貌集装箱物流中心建设，开工建设诚通西部物流中心，完成康心医药物流配送中心、瀑布冷链物流中心等项目建设，培育发展电子商务企业，新增3A级物流企业3家。加快发展金融业。加快贵阳国际金融中心建设，确保第一批金融单位入驻。继续实施“引金入筑”工程，促成民生银行、兴业银行、光大银行等贵阳分行挂牌开业。加快组建贵阳农村商业银行，推动贵阳银行跨区域经营工作。进一步优化金融生态环境，严厉打击非法集资等活动。组建大宗商品交易所、股权交易所，初步建立多层次、多品种的要素市场体系。加快发展会展业。围绕“做优会议业、做特展览业、做精节庆业、做强赛事业”要求，以国际会展中心为重要基地，精心筹办中医药博览会、亚洲青年动漫大赛、绿茶博览会、酒博会和特色农产品交易会、国际车展等50多个会展活动，打造中国夏季会展名城。发展壮大商贸流通业。完成孟关汽贸城、贵阳石板农产品批发市场一期工程，培育和打造辐射西南和全国的特色专业批发市场；积极推动龙博置地广场购物中心、中大国际广场、元隆广场等一批大型商业综合体建设，新建20个社区便民连锁超市、30个社区家政服务连锁店和50个以上放心粮油直销店，培育一批商贸流通业“亿元级企业”。推进农超对接，抓好家电、汽车、摩托车等下乡，促进消费繁荣。

加快发展现代农业，增强农业和农村发展活力。继续推动“畜、禽、蛋、奶、蔬、果、花、茶、药、烟”十大产业发展，抓好乌当、花溪国家农业科技示范园区、贵州省现代农业展示区和开阳、息烽农产品发展优势区建设，建立白云食用菌示范园区。完成三联公司乳品加工厂搬迁和4个标准化牧场建设，新增奶牛存栏1万头；加快公路沿线和25度以上坡耕地结构调整，集中连片打造20个500亩以上特色产业精品生产示范基地，新增蔬菜播面10万亩次、果树基地2万亩、茶叶基地1万亩、中药材基地3万亩。推进农业产业化经营，重点抓好台农公司20万头和特驱希望40万头高标准商品猪基地建设，新增产值上亿元龙头企业5家。组建市农业投资担保公司，扶持100个农民专业合作组织。推行生态农业循环模式，有机肥使用面达50%以上，促进农村能源多元化、农村生活清洁化、农业生产无害化。新增无公害农产品80个、绿色食品5个、有机农产品2个，农产品质量安全合格率达98%以上。加强农村基础设施建设。抓好农业综合开发土地整治项目，实施高标准基本农田建设和中低产田土改造6万亩。完成9座病险水库除险加固和150平方公里水土流失治理工程，解决35万农村人口的饮水安全问题。

（二）强力打好“三创一办”攻坚战，在提升基础设施、市容市貌、文明形象上求突破、见实效

按照“融入国际化、实现现代化、体现人文化、突出生态化”要求，以“三创”促进城市面貌、城市品质大提升，为协办民运会打造更靓丽、更整洁、更有序、更文明的环境；通过协办一届有特色、成功的民运会，促进城市管理水平、市民文明素质大提升，扩大知名度、影响力，推动“三创”工作上台阶、上水平。

加强城市建设，展现城市风采。加快城市“北拓、南延、西连、东扩”步伐。切实发挥城市总体规划、土地利用总体规划的引导调控作用，围绕“一城三带多组团”的格局，以城市综合体推动城市拓展，加快三马片区物流商贸带、龙洞堡食品轻工业园、贵州旅游商品城、乐湾国际温泉城、多彩贵州城等项目建设，促进三马片区、东部新城以及乌当、花溪、清镇等组团建设。新增城区面积20平方公里。成立棚户区城中村改造办公室，着力完善配套政策，有序推动旧城改造，加快老城区稀化、美化、绿化、亮化进程。金阳新区重点推进金融中心、西南物流城等重大项目建设的同时，围绕协办民运会抓好城市环境综合整治，加速与老城区融合发展。高标准加强枢纽型、功能性、网络化重大基础设施建设。以8月31日为时间节点，全面完成“两路二环”、西南环线二期、金清线等城市干道的主体及配套工程建设、景观绿化等工作。立足建设枢纽城市目标，加快完善城市对内对外交通体系建设，继续配合抓好贵阳至广州、重庆、成都、昆明、长沙的高速铁路及龙洞堡机场改扩建等项目建设，加快推进市域快速铁路、轻轨1、2号线建设、210国道三桥至沙文段改造，新建及改造文化山路网、金阳火车站功能区路网、改貌路、太慈桥片区路网、彭家湾片区路网、渔安安井片区路网等主次干道及支路。认真负责抓好44个涉及总投资78.29亿元建设项目的“投转固”工作。高起点推进民运会场馆及配套设施建设。按照“安全、质量、进度、功能、成本”五统一的原则，确保奥体中心主体育场、市民健身中心及其他比赛场馆成功测试、运行，抓好国际会议中心、接待酒店、新闻中心等相关设施的建设和交付使用。

注重市容市貌整治和氛围营造，提升城市形象。实施民运会城区添彩工程。编制并实施城市夜景规划，打造“一环路”以内各主干道和主要出入城通道沿线和南明河沿岸的亮丽走廊。实施城市景观升级工程，完成花溪大道、沙冲路、改茶路、百花大道、嘉润路等城区主干道、主要出入城通道及民运会场馆周边环境综合整治，鼓励企业、个人认捐、认种花木，实现四季常绿、四季有花，推动城区景观园林化。完善数字化城市管理系统，实现数字化城市管理全时段、全方位覆盖。开展“五在贵阳”推广活动。精心组织避暑季、温泉季、美食节、购物节等活动，促成一批体育公园、星级酒店、酒吧街开业，开发一批民俗街区、风情村镇、旅游产品，集中宣传和推广贵阳名景、名吃、名店，提高“游在贵阳、食在贵阳、住在贵阳、购在贵阳、乐在贵阳”的知名度。做好宣传推广工作。通过“走出去”、“引进来”，既遵循重大体育文化活动的国际传播惯例，也遵循城市形象的传播规律；既强化民族传统体育的活力，也强化协办城市的魅力；既展示多彩的民族风情，也展示独特的城市风情。以避暑季开幕为起点，陆续开展民族文化展示周活动繁荣文化创作、文化产业和城市精神文化生活。

做好服务保障，展现城市风尚。协办好“三大”活动。积极配合省安排好各项赛事，精心组织好开闭幕式、民族大联欢等三大活动，完善各项运营服务保障工作，确保办成一届特色、成功、团结的民运会。保障好城市运行。全力做好比赛期间对外宣传、交通通信、住宿餐饮、水电气供应、环境卫生、医疗卫生等工作，健全场馆外围保障与场馆运行的协调机制，提高应对极端天气和突发事件影响的快速反应能力，确保场馆运行和城市运行的无缝衔接。健全以属地管理为主的维护稳定工作体制，有效预防、依法严厉打击各类违法犯罪活动。开展“迎民运、讲文明、树新风”活动。广泛深入进行社会动员，增强“人人都是东道主”意识，尊重各民族的风俗习惯和宗教信仰，充分展示贵阳文明形象。全面加强志愿者招募、培训、管理和服务站点建设工作，深入开展志愿者服务活动，把志愿者队伍建成一道亮丽风景线。把提高窗口服务质量与便民利民、树立品牌相结合，建立健全窗口信息问讯系统，加强标识标牌、无障碍设施、刷卡消费等设施建设与日常维护，促进窗口行业服务水平整体提高。

（三）大力推进生态文明建设，在建设资源节约型、环境友好型城市上求突破、见实效

大力发展绿色经济、循环经济、低碳经济。积极开展低碳城市试点工作，认真实施低碳发展专项规划。重点推进开阳、息烽、清镇、修文循环经济生态工业基地建设，加快贵州磷都化工公司黄磷尾气综合利用年产甲酸（二期）、开阳青利天盟公司利用黄磷尾气延长产业链等项目建设，促进磷、铝资源就地转化。切实抓好城市LED照明系统、新能源汽车试点和公交LNG清洁燃料推广工作，建成一批低碳社区和小区。深入推进节水型城市建设，抓好高耗水行业的节水和废水资源化工作，健全并严格执行浪费水资源的螺旋式开关、马桶等生活建材的市场退出机制，使节水工作走在西部城市前列。

大力推进节能减排。坚决淘汰落后产能，严格控制高耗能、高污染产业，抓好清镇发电厂、贵钢、贵阳电厂等企业老厂搬迁工作。深入推进“两湖一库”治理和保护，确保饮水安全。开工建设二桥污水处理厂二期、花溪南部污水处理厂等项目，完善已建成城镇污水处理厂配套管网，开展城区河道治理及大沟改造工程。抓好大气和主要污染物减排工作，进一步改善空气质量，空气质量优良率达95%以上。

大力培育生态文化。认真谋划2011生态文明贵阳会议，推动会议机制化建设。深入推进生态文明社区（村）、学校、医院、企业、机关等创建工作，让生态文明观念在全社会牢固扎根。推动垃圾分类回收，倡导乘坐公交、使用绿色产品、减少一次性用品等绿色生活方式。

（四）着力推进改革开放和自主创新，在增强发展动力和活力上求突破、见实效

推进创新型城市建设。加快完善创新体系。加快重大科技平台建设，在新材料、高端装备制造、磷煤精细化工产业等领域建设一批产业技术创新联盟，建设药业研究院、特色生物科技产业工程技术研发中心等公共技术服务平台，加快建设酒类及加工食品、电子基础元器件、新型墙体材料、磷及磷化工产品等4个国家级质量监督检测中心。全面推进科技金融工作，力争成立1—2个创业投资机构，构筑3—4个科技贷款合作平台，推动科技企业到创业板或中小板融资。加大技术成果转化力度，重点支持高性能铝合金新材料制造工艺技术开发、工程机械液压多路阀、黄磷尾气提纯利用等重大项目实施。大力推进知识产权和品牌战略，深入开展打击侵犯知识产权和制售假冒伪劣商品专项行动，实施“百企知识产权培育工程”，力争1—2个产品获中国名牌、中国驰名商标称号。加强人才队伍建设。继续实施“高端人才引进计划”，力争引进1—2名行业领军人才和10名以上核心专家，培育、引进创新型适用人才，提升产业人才队伍整体素质，以人才结构的转型优化促进加速发展、加快转型，为建设创新型城市提供智力支撑。启动高新技术产业和战略性新兴产业千亿元行动计划。加快建设中航飞机发动机生产线、贵州皓天光电LED蓝宝石衬底材料、泛特尔白蛋白扩建等项目，促进战略性新兴产业加快发展。

扎实推进重点领域关键环节改革。进一步推进行政管理体制改革，及时总结城市基层管理体制改革试点工作经验，逐步推开“两级政府、三级管理”城市管理体制。认真抓好国家各项财税改革政策的落实，保持财政收入稳定增长。完善政府购买公共服务机制，通过招投标、委托服务、管理承包、特许经营等市场化手段，鼓励和支持社会力量参与城市运营等公共服务。深化市属国有控股企业改革，加强国企经营业绩考核，完善国有资产监管体制。组建地产集团，完善投融资平台公司风险预警与偿债机制，加快形成“融投建、借用还”一体化机制。

全面推进招商引资。围绕优势产业发展、产业结构升级，精心做好招商引资项目库建设，使招商引资项目库投资总额保持在3000亿元以上，招商引资引进资金、到位资金增长20%以上；整合招商资源，建立各级政府、园区、工商联、企业联动机制，形成招商合力；强化项目落地协调服务，实施项目分级推进责任及结果倒逼制度，推行重大项目投资全程专人代理服务制，大力营造“亲商、爱商、安商、敬商”的良好环境。瞄准重点产业中有重大影响和带动力的行业龙头企业，继续加大面向央企招商工作力度，采取增资扩股、收购兼并、产业链招商等引资方式，进行重点推介和专题招商，力促与央企签约大项目落地。扩大对外贸易规模。搭建功能齐全、政策优惠、手续简化的贸易平台，加快推进贵阳综合保税区和“无水港”建设，积极申报商务部中西部加工贸易重点承接地。积极培育对外贸易主体，鼓励企业开展自营出口业务，新增进出口总额上亿美元的企业1—2家。

支持非公有制经济和中小企业发展。放宽市场准入，坚持非禁即允，支持民间资本进入资源开发、基础设施、公用事业和金融服务业等领域，促进民营经济实现公平待遇。加快中小企业服务体系建设，完善中小企业服务平台。继续实施“小巨人企业成长计划”，鼓励企业通过兼并重组迅速发展壮大，通过银行贷款、发行债券、企业上市等解决融资瓶颈，力争2—4家企业上市，新增规模以上企业50家。非公经济增加值增长20%以上。

(五)全力实施“十大民生工程”，在建设和谐贵阳上求突破、见实效

围绕“十大民生工程”的五年目标和任务，分解年度目标和责任单位、责任人。市级财政民生支出比上年提高1个百分点。实施就业和增收工程。力争通过国家级创业型城市验收。推行更加积极的就业政策，鼓励扶持自主创业，多渠道开发就业岗位，强化就业援助。投放小额担保贷款8000万元，转移农村富余劳动力2.5万人，大力创建充分就业社区，继续保持“零就业”家庭动态为零。加强劳动执法，促进劳动关系和谐。实施扶贫济困工程。继续深入推进开发式扶贫，加大项目、资金和技术扶持力度，切实提高农村贫困人口自我发展能力。完成3万人减贫任务。进一步完善社会保障体系，实现基本医疗、失业、工伤保险分别扩面4万人，积极推进农民工参加社会保险，建立低保标准自然增长机制，建成三级城乡低保信息网络。全面加强物价监管，做好粮油、猪肉、蔬菜等主要农副产品的应急调控，实行价格补贴与物价上涨联动机制，做好水利水电工程移民、低保户、困难职工、大中专院校家庭经济困难学生等群体的价格补贴工作，确保低收入群众不因物价过快上涨而降低生活水平。实施普教优教工程。制定并实施《贵阳市2010—2020教育改革和发展规划》，提高教育现代化水平。设立学前教育发展专项资金，制定学前教育生均公用经费标准，大力发展公办幼儿园，进一步鼓励并规范民办学前教育发展，切实解决“入园难”问题。巩固提高义务教育质量和水平，深入推进学区化改革，在主城区新增2—4所标准化公办学校，用于进城务工人员子女接受义务教育，在乡镇政府所在地改扩建10—20所寄宿制标准化学校。力争创建1—2所国家级、省级示范性职业院校。完善教育资助制度，扶助家庭经济困难学生完成学业。实施健康工程。推进社区卫生服务体制机制改革，全面实施基本药物制度，建立完善居民健康档案，积极探索公立医院改革新路子，组建贵阳公共卫生救治中心，开工建设康复疗养基地、乐康国际医疗健康城等项目，促进区（县）二级甲等医院评审工作，开展“诚信医院”创建活动，规范医疗执业行为。切实加强人口计生工作，深入推进综合改革，保持全省领先地位。继续稳定低生育水平，提高出生人口素质，强化流动人口计生服务管理，完善覆盖城乡的计生公共服务体系，扎实抓好计生家庭“奖扶医帮优保”利益导向政策的落实到位。人口出生率控制在10.7‰以内。广泛开展全民健身运动，进一步提高竞技体育实力，力争选拔培养更多的运动员参加民运会，组团参加全国第七届城运会，举办“中国之队”国际足球赛等有影响力的体育赛事活动，建设黔灵山公园至开阳南江大峡谷国家级体育健身步道，打造生态健身示范公园，不断推进体育产业发展。实施养老敬老工程。完善城镇基本养老、新型农村社会养老保险制度，落实老年人生活补助制度，加快实现养老保险全覆盖。推进养老服务体系建设，逐步形成以居家养老为基础、社区服务与机构养老相配套、满足城乡多层次、多样化服务需求的养老服务网络。深化公立养老服务机构改革，鼓励社会力量兴办养老服务机构。实施安居工程。强化政府责任，调动社会各方面力量，进一步完善住房保障体系，重点抓好花果园、汉湘街、汤巴关、大营坡、河滨剧场等10个城中村、棚户区、旧城成片改造项目，促进房地产市场健康发展，努力解决各类群体的住房问题。实施平安工程。坚持用群众工作统揽信访工作，加大对基层矛盾纠纷的排查调处力度，不断完善信访工作体制机制，努力做好新形势下信访工作。加快建设公共安全视频信息系统，深入推进“严打”整治斗争和各项专项行动，依法打击“两抢一盗”、贩毒等犯罪，使群众安全感明显增强，满意度高于全国平均水平1个百分点以上。加强对消防、交通、煤矿、建筑、水利、危化、特种设备等重点行业和领域的安全生产常态化监管，实现生产安全事故起数和死亡人数“双降”。切实加强餐饮食品、药品、医疗器械、保健食品、化妆品的日常监管、专项整治和抽样检测，实施基本药物全品种电子监管和抽验，保障人民群众饮食用药安全。完善灾害性天气监测预警系统建设，提高气象灾害防御能力。继续深化应急工作“一案三制”建设，大力提升应急处置能力，高效处置突发事件，确保社会和谐稳定。实施畅通工程。强化交通执法管理、停车秩序管理，合理组织交通，提升交通事故、交通拥堵等突发事件的处置能力，提高路口通行能力，减少拥堵节点。坚持公交优先，加快公共交通设施建设，优化公共交通布局，引导公交服务向居民小区、城郊结合部以及农村延伸，提高公共交通的吸引力和分担率。坚持不懈打击“黑摩的”、“黑的士”，维护客运市场秩序。特别是民运会期间科学组织、合理实施交通管理措施，重点保障公共交通服务，力求实现赛时交通与城市日常交通和谐运转。实施生态建设和整脏治乱工程。严格执行“绿线”管理制度和绿地系统规划，继续实施石漠化综合治理、退耕还林和天然林保护工程，加大采石场、矿山等迹地的环境治理和土地复垦力度。确保森林覆盖率提高1个百分点，新增城区绿地40万平方米。建成危险废弃物暨医疗废物处置中心、南郊垃圾填埋场等市政基础设施项目。深入开展除尘降噪和整治乱搭乱建等专项行动，强化门前（摊前）“三包”责任制，进一步完善市容环境长效管理机制。实施乡村清洁工程，全面整治农村环境卫生。实施传统美德弘扬工程。促进广电中心、城市规划展览馆、市科技馆、市档案馆、市（金阳）图书馆、市文化遗产博物馆、阳明文化园等文化基础设施项目建设，继续推进乡镇文体中心、农家书屋、村文化室等建设，深入实施农村广播电视村村通工程。继续开展扫黄打非工作。繁荣文艺创作，推出《王阳明》等一批影视文艺作品。

（六）努力加强政府自身建设，在依法行政、提高效能、廉政建设上求突破、见实效

始终秉持为人民谋幸福的理念，按照“团结、务实、勤奋、廉洁”的要求，努力建设法治政府和服务型政府，为加速发展、加快转型、推动跨越提供有力保障。

切实推进依法行政。自觉接受市人大及其常委会的依法监督和市政协的政治协商、民主监督，认真执行各项决定决议，切实提高人大代表建议和政协提案办理工作质量。主动听取各民主党派、工商联、无党派人士和人民团体的意见建议。自觉接受新闻舆论和人民群众的监督。坚持公开是常态、不公开是例外，进一步增强公共政策透明度和市民参与度，深化政务、村务、厂务公开和公共企业、事业单位办事公开，凡涉及群众利益的重大事项都要向社会公开，进一步规范行政管理执法和行政行为，促进科学民主决策，提高政府公信力。

切实提高政府效能。以开展“项目建设年”、“作风建设年”、“环境建设年”和“四帮四促”活动为载体，着力建设效能政府。合理界定政府权责，全面推动政企、政资、政事、政府与市场中介组织分开，强化社会管理和公共服务，创新公共服务体制机制，提高公共服务质量和效率。切实增强为贵阳发展“坐不住”、“等不起”、“慢不得”的责任感、紧迫感，加强政府机关效能建设和精细化管理，推行行政问责、绩效管理、服务承诺、首问责任、限时办结、绿色通道等制度，进一步精简会议、文件，规范检查评比达标活动，确保工作精力集中到抓项目、抓产业、抓发展上来，确保政府系统执行力和服务水平有明显提升。

切实加强和创新社会管理。进一步强化政府社会管理职能，大力培育社会组织，广泛动员和组织群众依法参与社会管理，以社区为载体，以市民需求为导向，进一步整合社会管理职能和服务资源，实现政府行政管理与群众自治有效衔接和良性互动，提升社会治理水平。大力加强国防后备力量建设，切实做好“双拥”工作。

切实加强廉政建设。认真贯彻落实领导干部廉洁从政若干准则，强化廉洁从政制度的执行监督。继续强化预算管理，落实厉行节约措施，行政公用经费支出保持“零增长”，公务接待、公务用车、出国（境）经费比上年下降5%。加大监察、审计工作力度，加强对群众反映强烈的突出问题的专项治理，加大查办腐败案件力度，以更加坚定的决心和更加有力的举措坚决惩治腐败、有效预防腐败，进一步提高反腐倡廉建设科学化水平。

各位代表，过去五年，我们谱写了贵阳发展史上的辉煌篇章；未来五年，我们肩负着加速发展、加快转型、推动跨越的光荣使命。让我们紧密团结在以胡锦涛同志为总书记的党中央周围，全面贯彻落实党的十七届五中全会、省委十届十次全会、市委八届十次全会精神，在省委、省政府和市委的正确领导下，高举发展、团结、奋斗的旗帜，深入贯彻落实科学发展观，践行“知行合一、协力争先”的贵阳精神，解放思想、振奋精神、开拓创新、真抓实干，为实现我市国民经济和社会发展第十二个五年规划，加快推进经济社会发展的历史性跨越而努力奋斗！
